# High-resolution dataset for building energy management systems applications

**DOI:** 10.1016/j.dib.2017.12.058

**Published:** 2018-01-03

**Authors:** Wessam El-Baz, Johannes Honold, Lukas Hardi, Peter Tzscheutschler

**Affiliations:** Institute for Energy Economy and Application Technology, Technical University of Munich, Germany

## Abstract

Modelling and optimization of energy management systems (EMS) require different data types for operation and validation. In this article, a multi-purpose dataset is provided for EMS applications. It includes PV measurement data for the PV generation and prediction algorithms associated with EMS systems. Weather data has also been measured at the same location for the optimization of PV prediction algorithms and other applications such as building model simulations. Moreover, the dataset contains detailed measurements of a seminar room where not only temperatures have been measured, but also user feedback for comfort assessment. All documented measurements have been gathered at the same location in Munich, Germany.

**Specifications table**

**Table:** 

Subject area	*Energy*
More specific subject area	*Renewable energy generation, energy management systems*
Type of data	*CSV files with different measurements (PV system, weather, occupancy)*
How data was acquired	*Data was acquired using two techniques (direct inverter reading, sensors attached to Raspberry Pis or Arduinos)*
Data format	*Raw data*
Experimental factors	*No*
Experimental features	*Very brief experimental description*
Data source location	*Munich, Germany (Theresienstraße 90, Building N8)*
Data accessibility	*The data is publicly accessible on*http://www.smartup.ei.tum.de/aktuelle-messungen/download/

**Value of the data**

• Available PV measurements are necessary for testing demand-side management algorithms
• Weather data at same location is available in order to assess heat demand of buildings, COPs of heat pumps etc.
• Smart seminar room data is valuable to evaluate the comfort of the occupants based on ambient temperature and heating system behavior
• Having all the data measured at high resolution in the same exact location makes it an ideal candidate for EMS applications

## 1 Data

All the data have been collected at this location: Technical University of Munich, Theresienstraße 90, 80333 Munich, Germany. On [1], live readings can be monitored and downloaded with different temporal resolutions for different periods.

**PV Measurements:**

Available PV measurements provide the following data:

• AC power
• AC voltage
• AC current
• Frequency
• DC power
• DC voltage
• DC current
• Energy generated today
• Energy generated throughout the system lifetime
• Inverter temperature

**Smart Seminar Room**

The room measurement sensors provide the following data:

• Temperatures at two locations (near windows, near doors)
• Humidity
• Four heating system valve actuator positions
• Number of occupants
• Daily feedback from the occupants on the room comfort

**Weather Station**

The weather station provides the following data:

• Temperature
• Humidity
• Atmospheric pressure
• Wind speed
• Wind direction

## 2 Experimental design, materials and methods

### 2.1 PV system measurements

**Fig. 1:**
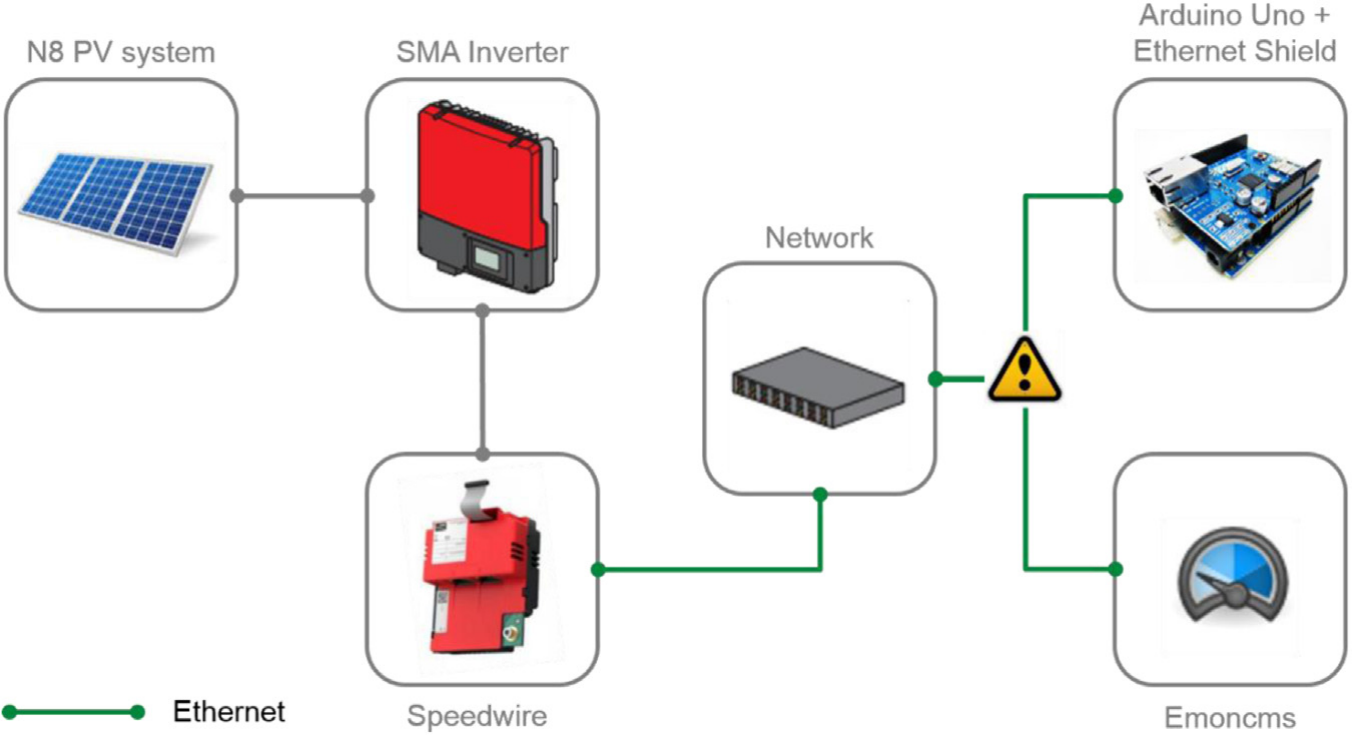
Data communication between the PV system and the energy monitoring database (Emoncms).

The system is a 3 kWp roof-top PV system that consists of 2 arrays, where each of which has six modules. Array 1 has monocrystalline modules (Solarworld SW 260 Mono), while array 2 has polycrystalline (Solar World SW 250 Poly). The arrays are connected to an SMA inverter Sunnyboy TL3000. The measurements are received directly via a UDP connection with the inverter as per Fig. 1 and fed to the database.

### 2.2 Smart seminar room

**Fig. 2:**
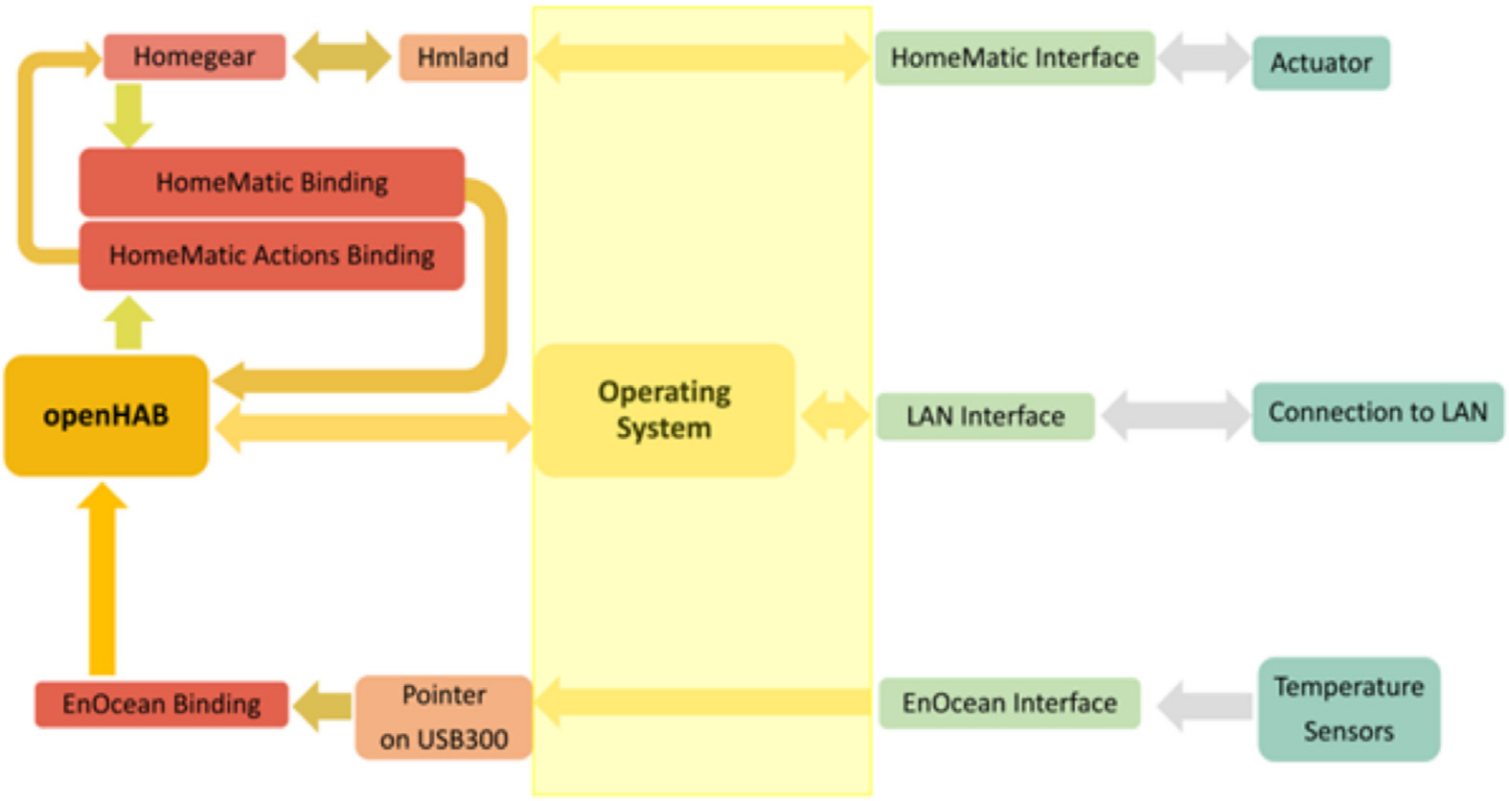
Overview of the measurement system [1].

Temperatures and humidity in the room are measured via EnOcean wireless sensors. The sensors send the data to a USB300 dongle attached to a Raspberry Pi, which represents the data collector and the EMS of the seminar room. The Raspberry Pi sends back control signals to the heating system actuators to open the heating valves at specific positions. Finally, it uploads all the data to the online database. Fig. 2 represents an overview of the control system.

**Fig. 3:**
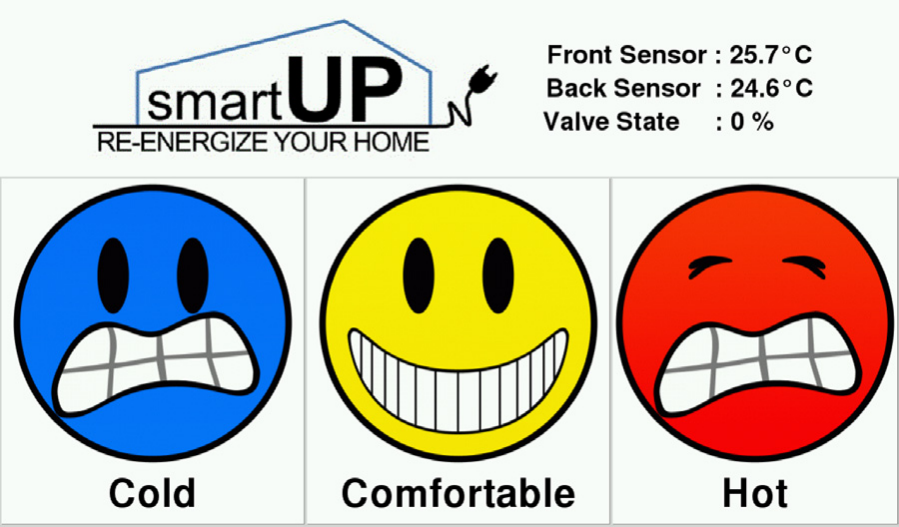
The feedback touchscreen interface.

The room occupancy is measured through a face detection camera based on a Raspberry Pi that counts the number of faces in the room. The feedback touchscreen has the GUI interface shown in Fig. 3, where the occupants can report on the room temperature at any time by clicking either on “cold”, “comfortable” or “hot”.

### 2.3 Weather station

**Fig. 4:**
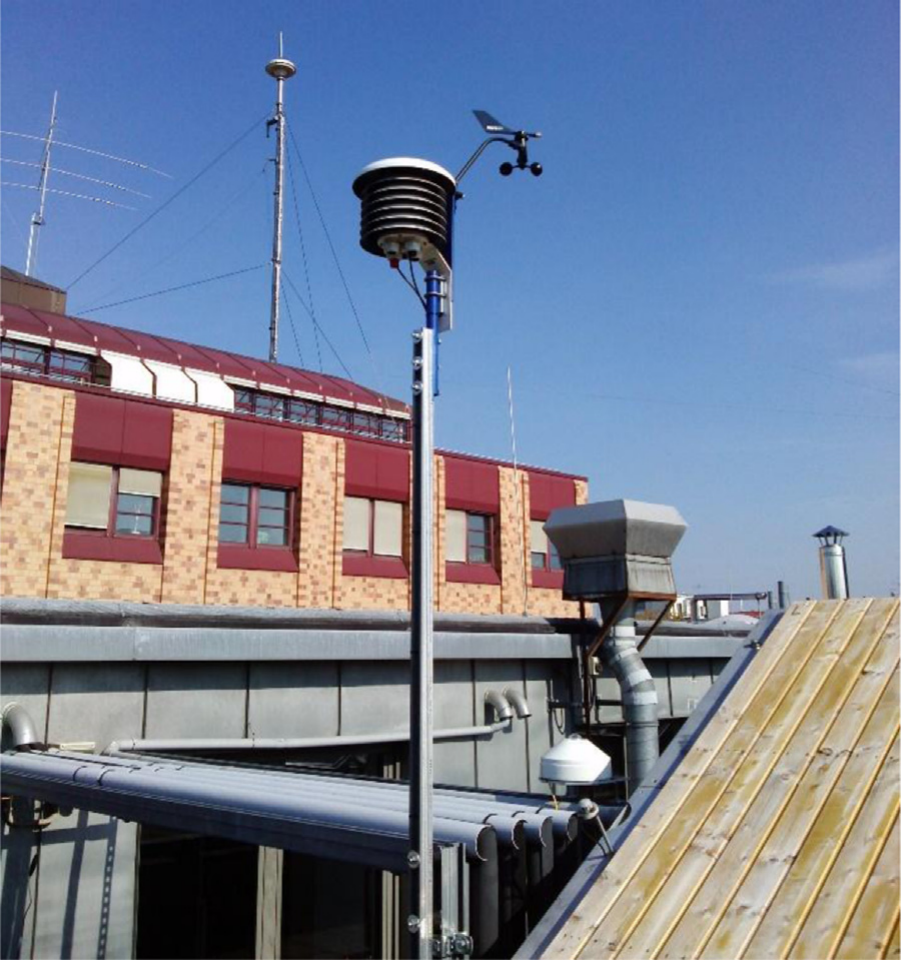
Weather station on the roof top.

The weather data is gathered on top of the building containing the smart seminar room. The sensors are mounted approx. 15 m above the ground on a trough-like roof of a four-story building on top of a metal pole in a passive cooled housing. A six-story building is located in 10 m air-line distance overshadowing the weather station (see brick building in Fig. 4).

The data about temperature, humidity, atmospheric pressure and wind speed is acquired by an Arduino Uno in 5 s intervals. The sampling rate measuring the wind direction is also at 5 s, but every 10 min the mean value is taken and stored. The pressure is converted to the mean sea level pressure.

The following sensor types are used to acquire the data

• Temperature: HTU21D, protocol: I2C
• Humidity: HTU21D, protocol: I2C
• Pressure: MPL3115A2, protocol: I2C
• Wind speed and direction: Davis 6410
